# Successful Lung Transplantation after Plasma Exchange in a Patient with High Thermal Amplitude Cold Agglutinins: A Case Report

**DOI:** 10.70352/scrj.cr.26-0502

**Published:** 2026-08-27

**Authors:** Yuya Miyoshi, Kentaroh Miyoshi, Naomi Asano, Haruchika Yamamoto, Shin Tanaka, Shuji Okahara, Mikio Okazaki, Seiichiro Sugimoto, Nobuharu Fujii, Shinichi Toyooka

**Affiliations:** 1 Department of General Thoracic Surgery, Okayama University Hospital, Okayama, Okayama, Japan; 2 Division of Transfusion and Cell Therapy, Okayama University Hospital, Okayama, Okayama, Japan; 3 Organ Transplant Center, Okayama University Hospital, Okayama, Okayama, Japan; 4 Department of Anesthesiology and Resuscitology, Okayama University Hospital, Okayama, Okayama, Japan; 5 Department of Hematology, Okayama University Hospital, Okayama, Okayama, Japan

**Keywords:** cold agglutinins, thermal amplitude, lung transplantation, plasma exchange, extracorporeal circulation, perioperative management

## Abstract

**INTRODUCTION:**

Cold agglutinins (CAs) are immunoglobulin M autoantibodies that induce red blood cell agglutination at low temperatures and may cause hemolytic or thrombotic complications during procedures associated with hypothermia. In lung transplantation (LT), blood may be exposed to low temperatures within the reperfused lung graft and the extracorporeal circulation circuit, potentially increasing the risk of CA-mediated complications. However, the perioperative significance of CAs in this setting remains unclear, and optimal management has not been established. We report a case of successful bilateral LT in a patient with high-thermal-amplitude CAs managed with preoperative plasma exchange (PLEX) and strict intraoperative temperature control.

**CASE PRESENTATION:**

A 55-year-old man with diffuse panbronchiolitis and secondary pulmonary hypertension was listed for bilateral LT. Preoperative testing incidentally revealed clinically significant CAs, with a titer of 1:128 at 4°C and a thermal amplitude of 30°C. Because intraoperative blood temperature might approach this threshold despite active warming, a single preoperative session of PLEX using 36 units of fresh frozen plasma was performed to reduce the risk of a CA-mediated complication. Bilateral LT was subsequently completed under meticulous temperature management, including extracorporeal circulation maintained at 37.5°C, continuous systematic warming, avoidance of topical ice application, and rewarming of the donor lungs before reperfusion. After surgery, the CA titer decreased to 1:32. The postoperative course was uneventful, without thromboembolic events, primary graft dysfunction, or bronchial complications.

**CONCLUSIONS:**

LT may be performed safely in selected patients with high-thermal-amplitude CAs when careful perioperative temperature control is combined with preoperative PLEX. PLEX should be considered when the anticipated intraoperative temperature is expected to approach the patient’s thermal amplitude threshold.

## Abbreviations

CA
: cold agglutinin
CAD
: cold agglutinin disease
DAggT
: direct agglutination testing
DTT
: dithiothreitol
ECMO
: extracorporeal membrane oxygenation
IgG
: immunoglobulin G
IgM
: immunoglobulin M
IVIg
: intravenous immunoglobulin
LDH
: lactate dehydrogenase
LT
: lung transplantation
PGD
: primary graft dysfunction
PLEX
: plasma exchange

## INTRODUCTION

CAs are IgM autoantibodies that induce red blood cell agglutination and hemolysis on exposure to low temperatures.^1)^ In CAD, agglutination may impair peripheral circulation, whereas hemolysis is primarily mediated by complement activation and may persist even after rewarming because complement remains bound to the erythrocyte surface.^2)^ However, CAs requiring clinical intervention as CAD are relatively uncommon.^3)^

In cardiac surgery, the clinical relevance of CAs has long been recognized because hypothermia during cardiopulmonary bypass may trigger circuit-related adverse events associated with hemagglutination and hemolysis.^4)^ By contrast, the clinical significance of CAs in LT has received limited attention.^5)^ Nevertheless, LT surgery may also expose blood to low temperatures through graft reperfusion (rewarming process) and the extracorporeal circulation procedure, increasing the risk of CA-mediated complications in patients with high-thermal amplitude.

We herein report a case in which clinically significant CAs demonstrating agglutination at room temperature were identified during preoperative evaluation for LT. The patient was managed with preoperative PLEX followed by strict intraoperative temperature control and eventually underwent successful bilateral LT. This case highlights important considerations in the management of LT recipients with CAs.

## CASE PRESENTATION

**Fig. 1:**
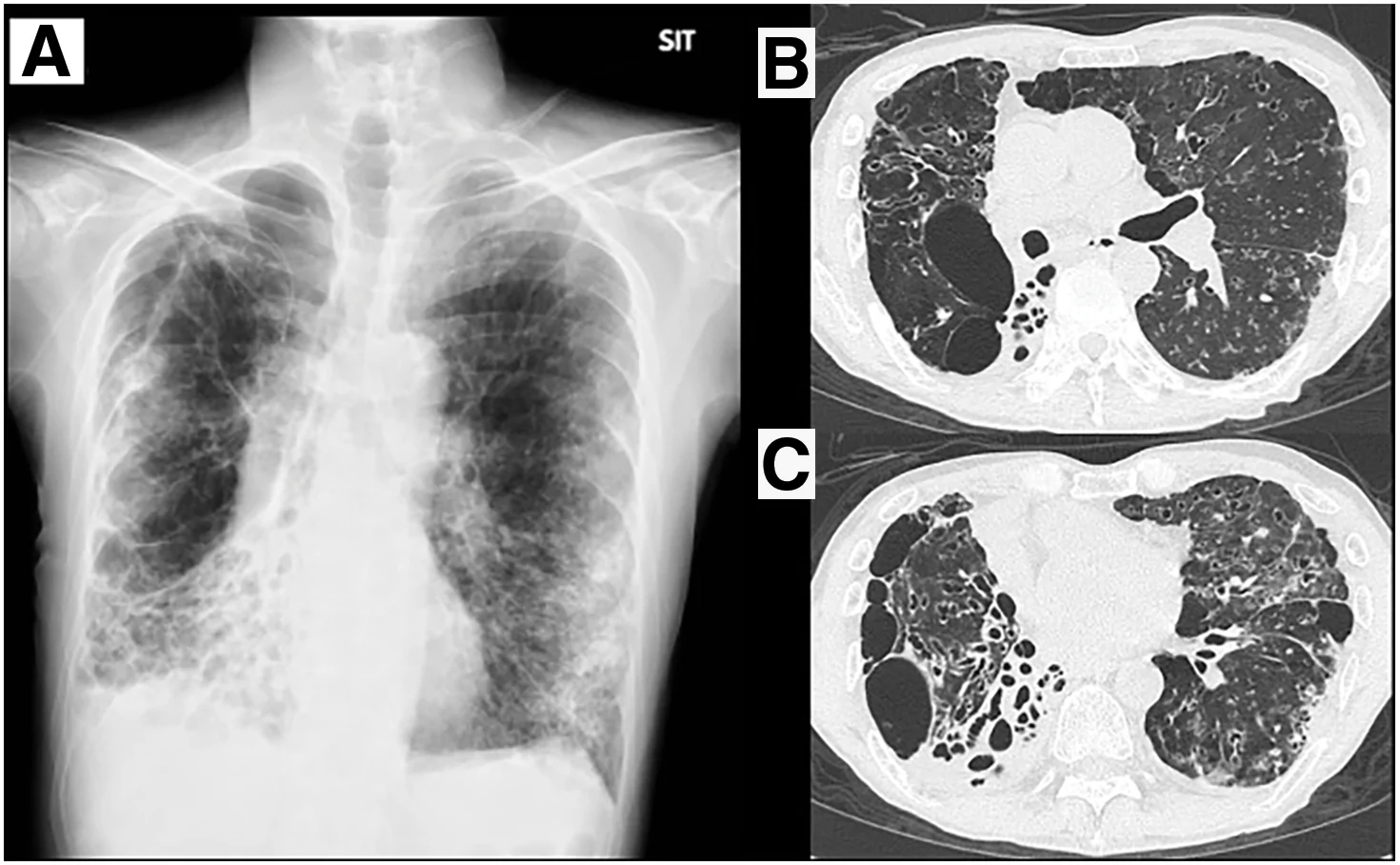
Chest radiograph (**A**) and CT (**B**, **C**) showing bilateral reticular opacities, centrilobular nodules, bronchiectasis, and bronchial wall thickening consistent with diffuse panbronchiolitis.

A 55-year-old man with diffuse panbronchiolitis and secondary pulmonary hypertension was listed for bilateral LT (**Fig. 1**). His clinical course had been complicated by recurrent bacterial pneumonia caused by multidrug-resistant *Pseudomonas aeruginosa* and a right pneumothorax treated with pleurodesis. Despite supplemental oxygen at 5 L/min via nasal cannula, arterial blood gas analysis demonstrated hypercapnic respiratory failure, with a partial pressure of oxygen of 110.4 mmHg and carbon dioxide of 69.2 mmHg. Transthoracic echocardiography showed a tricuspid regurgitation pressure gradient of 47.9 mmHg, consistent with secondary pulmonary hypertension. At the time of transplantation, he had severe cachexia, with a BMI of 15.7 kg/m^2^, and markedly impaired functional capacity. Laboratory testing showed a red blood cell count of 3.56 × 10^6^/μL, hemoglobin of 8.7 g/dL, LDH of 208 U/L, and total bilirubin of 0.29 mg/dL. Although anemia was present, there was no laboratory evidence of hemolysis.

**Fig. 2:**
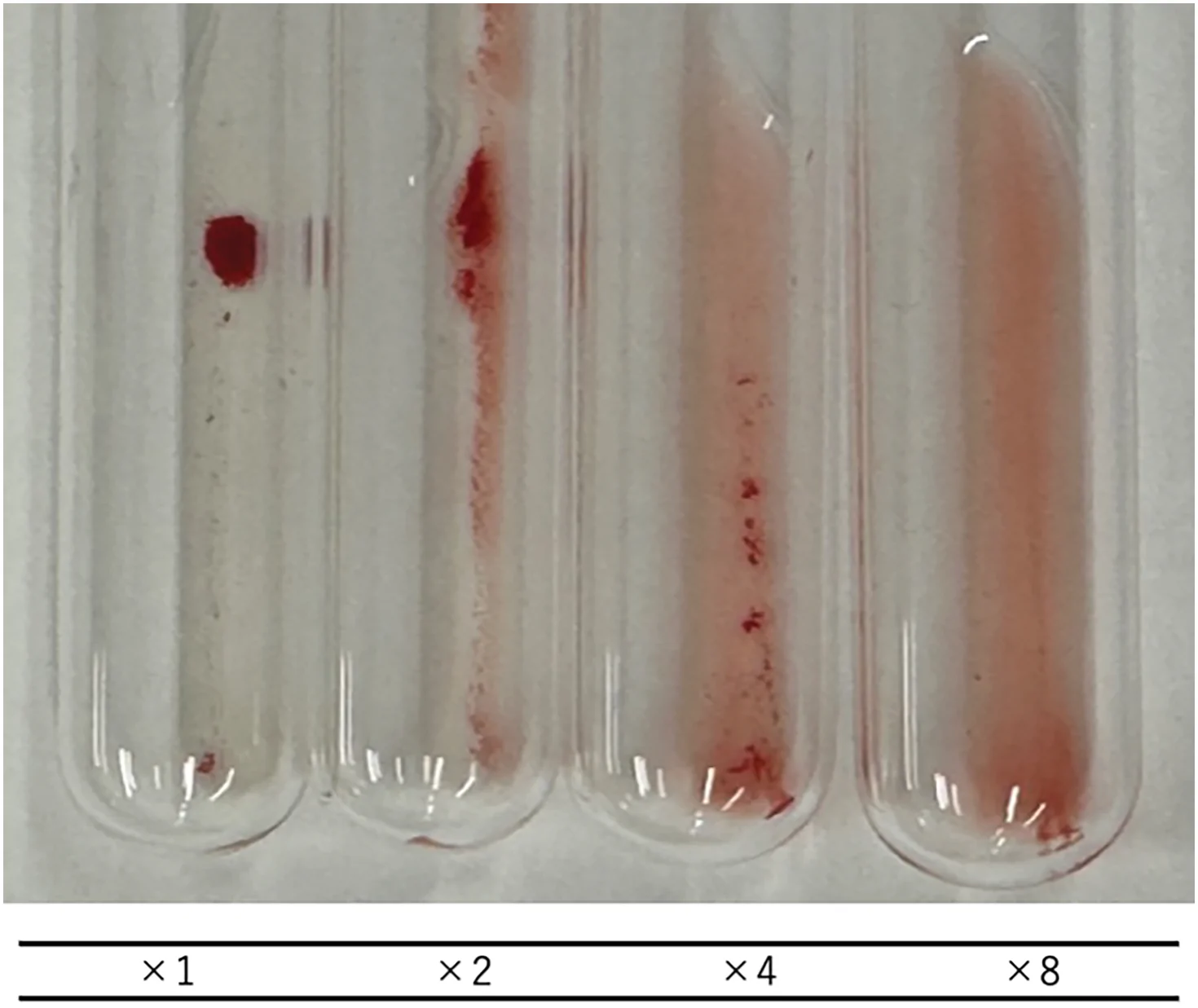
Patient plasma was serially diluted and tested with 3%–5% type O red blood cells after 60-minute incubation at room temperature. At a dilution of 1:4, agglutination was graded as 1+, and at 1:8, a weak positive (W+) reaction was observed, indicating barely visible microscopic agglutination.

Pretransplant lymphocyte crossmatching with the donor revealed positivity for both T and B cells; however, the crossmatch became negative after DTT treatment, and organ allocation proceeded. On the day before surgery, routine pretransplant workup revealed unexpected serologic abnormalities. Forward typing demonstrated blood group A. Reverse ABO typing at room temperature (24.2°C) showed agglutination of the patient’s plasma with both type A and type B reagent red blood cells, and indirect antiglobulin testing at 37°C demonstrated agglutination with all screening cells. These findings suggested the presence of an antibody with broad red blood cell reactivity. Further investigation identified an elevated CA titer of 1:128 at 4°C (reference, <1:32) with visible agglutination persisting at 30°C (**Fig. 2** and **Table 1**), indicating high-thermal-amplitude CAs. Direct antiglobulin (Coombs) testing was positive for IgG and negative for complement binding. Agglutination reactions were graded and converted to a titration score according to the method described in the Association for the Advancement of Blood & Biotherapies (AABB) Technical Manual.^6)^ The CA titer was defined as the highest dilution showing 1+ agglutination. Although the patient’s CAs showed minimal agglutination at 37°C, this finding raised concern for potential perioperative complications (**Table 1**).

**Table 1:** Preoperative and postoperative direct agglutination test results

A. Preoperative											
Temperature	×1	×2	×4	×8	×16	×32	×64	×128	×256	×512	Score
4°C	4+	4+	4+	4+	4+	3+	2+	1+	W+	0	85
Room temperature (24.2°C)	3+	2+	1+	W+	0	0	0	0	0	0	25
30°C	1+	W+	0	0	0	0	0	0	0	0	7
37°C	W+	0	0	0	0	0	0	0	0	0	2
B. POD 9											
Temperature	×1	×2	×4	×8	×16	×32	×64	×128	×256	×512	Score
4°C	4+	4+	3+	3+	2+	1+	W+	0	0	0	59
Room temperature (24.2°C)	1+	0	0	0	0	0	0	0	0	0	5
30°C	W+	0	0	0	0	0	0	0	0	0	2
37°C	0	0	0	0	0	0	0	0	0	0	0

Direct agglutination test results at various temperatures showing a CA titer of 1:128 at 4°C, with thermal amplitude extending to 30°C (underlined) preoperatively (A). Thermal amplitude is defined as the highest temperature at which CA-induced agglutination is observed. Postoperatively, the titer decreased to 1:32, and the thermal amplitude was reduced to room temperature (24.2°C) (underlined) (B). Agglutination reactions were graded and converted to score values according to the AABB Technical Manual (4+ = 12, 3+ = 10, 2+ = 8, 1+ = 5, W+, as defined in **Fig. 2**, = 2, 0 = 0). The titration score was calculated as the sum of scores across all dilutions tested.

AABB, Association for the Advancement of Blood & Biotherapies; CA, cold agglutinin

Given the broad thermal amplitude of the CAs, we convened a multidisciplinary discussion involving the lung transplant surgeons, anesthesiologists, perfusionists, and hematologists to assess the risk of red blood cell agglutination and microvascular thrombosis during LT surgery. Because blood within the ECMO circuit and the reperfused lung grafts could be exposed to temperatures approaching the patient’s thermal threshold of around 30°C, we judged that preemptive intervention was necessary to minimize the risk of thrombotic complications and graft injury.

Before induction of general anesthesia, a single session of PLEX was performed to reduce circulating IgM levels. A total of 36 units of fresh frozen plasma (approximately 4.3 L) were used as the sole replacement fluid to maintain adequate coagulation factor levels in anticipation of the subsequent surgical procedure. Nafamostat mesylate was administered as the anticoagulant (100 mg loading dose, followed by 20 mg/h maintenance). All replacement fluids were warmed to 37°C prior to administration to minimize cold exposure. The procedure was completed in 150 minutes. PLEX was completed at 14:00, general anesthesia was induced at 15:30 (90 minutes after completion of PLEX), and graft reperfusion was performed at 22:35, approximately 8 hours 35 minutes after completion of PLEX. Subsequently, bilateral sequential LT was performed under central venoarterial ECMO support. Meticulous temperature management was implemented throughout the procedure to avoid cold exposure. In addition to standard warming of intravenous fluids and blood products and use of a forced-air warming blanket, the ECMO circuit was actively maintained at 37.5°C. In contrast to our usual practice, an ice slush bag was not placed in the thoracic cavity during vascular anastomosis. Instead, immediately before reperfusion, the thoracic cavity and donor lungs were actively rewarmed by intrathoracic lavage with warm saline (36°C) to minimize the risk of CA-induced agglutination during reperfusion. Operative time was 9 hours 22 minutes, with the duration of ECMO support of 267 minutes. Ischemic times were 534 minutes for the right lung and 650 minutes for the left lung. The minimum intraoperative core temperature (bladder) and skin temperature were 37.1°C and 34.8°C, respectively.

**Fig. 3:**
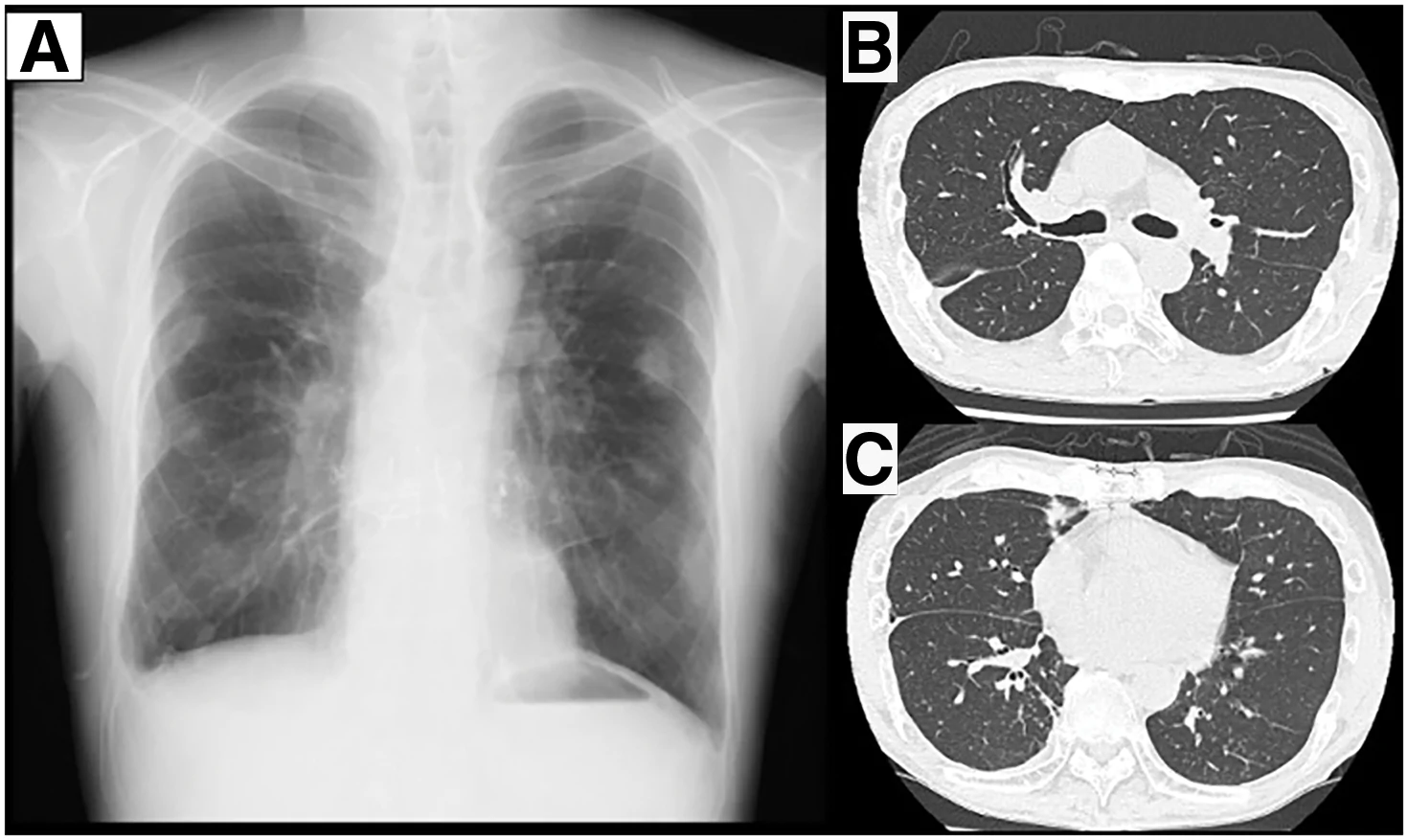
Chest radiograph (**A**) and CT (**B**, **C**) at hospital discharge showing well-expanded bilateral lung grafts without any graft-related complications.

Postoperatively, we closely monitored hemolysis markers, including LDH and bilirubin, and thrombotic complications as well as the coagulation marker D-dimer. Although systemic heparinization was administered intraoperatively as part of the ECMO protocol (activated clotting time target: 160–180 seconds), no additional anticoagulation therapy was administered postoperatively for the prevention of CA-related thrombosis. The immunosuppressive regimen consisted of tacrolimus, mycophenolate mofetil, and corticosteroids, with basiliximab used for induction. PGD was grade 0 at 72 hours after LT, and there was no evidence of hemolysis, thromboembolic events, or bronchial complications thereafter. Repeat testing on POD 9 revealed a decreased CA titer of 1:32 at 4°C and that the thermal amplitude had fallen to room temperature (24.2°C) (**Table 1**). Although tracheostomy was required to facilitate rehabilitation because of preexisting muscle weakness, the patient was successfully weaned from the ventilator on POD 39 and discharged on day 90 without a requirement of supplemental oxygen (**Fig. 3**).

## DISCUSSION

According to the 2017 diagnostic algorithm for autoimmune hemolytic anemia published by the British Society for Haematology,^7)^ cases with positivity for IgG on direct antiglobulin testing are suggestive of warm autoimmune hemolytic anemia, whereas those with positivity for C3d, with or without IgG, warrant further evaluation for CAD through DAggT at room temperature. A diagnosis of CAD is supported when DAggT is positive and the CA titer is at least 64. In the present case, although the patient showed an atypical direct antiglobulin test pattern, with positivity for IgG but not complement factor. Nevertheless, DAggT was observed up to 30°C, and the CA titer was 128. We therefore concluded that these CAs were clinically significant.

Although low-titer CAs can be detected in healthy individuals, clinically significant CAD requiring clinical intervention is uncommon.^8)^ In cardiac surgery, CAs have been detected in a variable portion of screened patients, with reported prevalence ranging from less than 1% to approximately 9%,^4,9)^ depending on the study population, screening methodology, and threshold used for positivity. In LT, Moneke I, et al.^5)^ reported that among 174 LT recipients, 78 (45%) had detectable CAs, and their presence were significantly associated with PGD Grade 2/3 and early postoperative thrombosis. However, 88% of detected CAs were reactive only at 4°C and were not associated with impaired long-term survival, suggesting limited clinical relevance in the absence of a broad thermal amplitude. By contrast, the 2 patients with reactivity at 37°C had catastrophic postoperative courses. One died immediately postoperatively, whereas the other developed multiple thrombotic complications and died after a prolonged ICU stay. These observations suggest that CAs with high-thermal amplitude are likely to be an important perioperative risk in LT. The same report described cases in which PLEX before LT resulted in favorable outcomes. Reports from cardiac surgery have similarly suggested associations between high-thermal amplitude and adverse events.^10–12)^ These findings informed our decision to prioritize thermal amplitude in operative risk stratification.

At our institution, routine screening for CAs is not performed before LT. Standard pretransplant testing includes ABO blood typing with forward and reverse grouping and indirect antiglobulin testing for antibody screening, followed by further antibody identification when a clinically relevant antibody is detected. Under this workflow, clinically insignificant CAs are unlikely to be detected. In the present case, however, reverse ABO typing and antibody screening demonstrated pan-agglutination at room temperature, prompting additional investigation that confirmed a clinically significant CA.

After its detection, we selected PLEX to achieve rapid reduction of circulating IgM before surgery. IVIg has been reported as a possible alternative and may theoretically mitigate risks related to vascular access and extracorporeal circulation during PLEX.^9)^ Corticosteroids might also be considered in selected situations,^13)^ although their efficacy in classic CAD is limited and the unpredictable timing of LT makes prolonged preoperative therapy impractical. In the present case, the CA titer decreased by POD 9. However, CA titer and thermal amplitude were not measured between the completion of PLEX and the initiation of surgery, and this reduction does not necessarily reflect the immediate effect of PLEX alone, as postoperative immunosuppression including corticosteroids, perioperative transfusions, and other perioperative factors may have contributed. Although PLEX is theoretically expected to reduce circulating IgM levels by direct removal, it remains unclear to what extent a single session of PLEX achieved sufficient antibody reduction in this case. Therefore, the precise effect of PLEX on CA levels cannot be determined from this case alone. Nevertheless, we believe that the decision to perform PLEX was a prudent safety measure, given the concern that intraoperative warming techniques alone might not have been sufficient to reliably prevent agglutination-related complications in a patient with such a broad thermal amplitude. The case has several important implications. First, incidental identification of CAs during pretransplant testing provided critical information for perioperative planning. Second, a management strategy combining PLEX with meticulous avoidance of cold exposure may have prevented CA-mediated complications in a patient with high-thermal amplitude. Third, timely multidisciplinary decision-making within the time window allowed for appropriate perioperative management.

## CONCLUSIONS

This case demonstrates that LT can be successfully performed after preoperative PLEX in a patient with high-thermal amplitude CAs reactive at 30°C. In such patients, meticulous perioperative management, including strict avoidance of cold exposure, may help prevent thrombotic complications and graft dysfunction. Accumulation of similar cases may contribute to refining risk assessment and perioperative management strategies in LT.

## DECLARATIONS

### Availability of data and materials

All datasets supporting the conclusions of this article are included in the manuscript.
